# Editorial Notes

**Published:** 1914-12

**Authors:** 


### Editorial Notes.

**Consulting Surgeon, Southern Command.**

Professor James Swain, M.D., M.S. Lond., Senior Surgeon, Bristol Royal Infirmary, has been appointed by the War Office as Consulting Surgeon to the troops serving in the Southern Command, with the rank of Lieutenant-Colonel. The duty involves Colonel Swain holding himself in readiness for consultation in cases of especial difficulty over the South and West of England, comprising the area west of Weymouth, Dorchester, Salisbury, and as far north as Wantage and Oxford, and with the large number of transfers from the expeditionary force, as well as the new armies now training in the Southern Command, his task can be no sinecure. We, however, feel it a legitimate cause for congratulation that the surgeon for such a very responsible office has been appointed from the Bristol School. Lt.-Col. A. E. J. Barker, F.R.C.S., R.A.M.C.T., has been appointed as Consulting Surgeon for the Eastern portion of the Southern Command. The corresponding appointments for the other commands hitherto made comprise Sir Arbuthnot Lane, Aldershot Command; Sir Frederic Eve, Eastern Command; Sir Charles Ball and Sir Thomas Myles, Irish Command.

**Second Southern Hospital Ambulance Work.**

The large numbers of British and Belgian wounded from the front sent for treatment at the Second Southern General Hospital have thoroughly tested the transport arrangements in connection with this Hospital, and the work has been none the lighter from the fact that this Hospital unit comprises three separate hospitals, each lying at a distance of about three miles from the other two. Nevertheless, all difficulties have been removed by the excellent organisation of the Bristol Red Cross Society and the St. John Ambulance Brigade, the transport work of both being under the direction of one head, the Bristol Red Cross Society's County Director, John S. Griffiths, being also Deputy Commissioner in charge of the Southern and Western District of the St. John Ambulance Brigade.

One may judge of the task imposed in that the removals have averaged over one thousand patients a month since the war began. They have fourteen ambulance wagons in use, the Bristol Tramways and Carriage Company have sent many motors, and a large number of private motor cars have been freely placed at the disposal of the transport organisation. They meet all trains, and frequently these arrive between midnight and one o'clock, and in this way during the week from December 26th to January 2nd no less than 460 wounded have been met at the railway station. Here the members of the women's detachments have done good service in supplying coffee and refreshments while the medical officers and orderlies are arranging the removal from train to ambulance. The Second Southern Hospital must be warmly congratulated on the assistance to their work which has been so well afforded in this way in the silent watches of the night.

